# Propofol Inhibits Microglial Activation *via* miR-106b/Pi3k/Akt Axis

**DOI:** 10.3389/fncel.2021.768364

**Published:** 2021-10-28

**Authors:** Jianhui Liu, Pu Ai, Yiyan Sun, Xiaoyu Yang, Chunhong Li, Yihan Liu, Xiaohuan Xia, Jialin C. Zheng

**Affiliations:** ^1^Department of Anesthesiology, Tongji Hospital Affiliated to Tongji University School of Medicine, Shanghai, China; ^2^Wuxi Clinical College of Anhui Medical University, Hefei, China; ^3^Center for Translational Neurodegeneration and Regenerative Therapy, Shanghai Tenth People’s Hospital Affiliated to Tongji University School of Medicine, Shanghai, China; ^4^Department of Cardio-Pulmonary Circulation, Shanghai Pulmonary Hospital, Tongji University School of Medicine, Shanghai, China; ^5^Translational Research Institute of Brain and Brain-Like Intelligence, Shanghai Fourth People’s Hospital Affiliated to Tongji University School of Medicine, Shanghai, China; ^6^Collaborative Innovation Center for Brain Science, Tongji University, Shanghai, China

**Keywords:** propofol, mir-106, microglia, neuroinflammation, lipopolysaccharide, Pi3k/Akt signaling

## Abstract

Propofol is an established intravenous anesthetic agent with potential neuroprotective effects. In this study, we investigated the roles and the underlying mechanisms of propofol in inhibiting the pro-inflammatory responses of microglia. Propofol significantly reduced the messenger RNA (mRNA) levels of *Tnf*, *Nos2*, and NF-κB pathway related genes *Ticam1*, *Myd88*, *Irf3*, and *Nfkb1* in lipopolysaccharide (LPS)-treated primary microglia. After screening the miRNA profiles in microglia under LPS and propofol treatment conditions, we found propofol abrogated the LPS-induced misexpression of miRNAs including miR-106b, miR-124, miR-185, and miR-9. Perturbation of function approaches suggested miR-106b as the core miRNA that mediated the anti-inflammatory effects of propofol on microglial activation. RNA sequencing (RNA-seq) analysis further identified Pi3k/Akt signaling as one of the most affected pathways after miR-106b perturbation of function. The treatment of Pi3k/Akt signaling agonist 740Y-P elevated miR-106b-reduced Akt phosphorylation and abolished the inhibitory effects of miR-106b on the pro-inflammatory responses of microglia. Our results suggest propofol inhibits microglial activation *via* miR-106b/Pi3k/Akt axis, shedding light on a novel molecular mechanism of propofol-mediated immunomodulatory effects and implying propofol as potential therapeutics for treating neuroinflammation-related neurodegenerative diseases.

## Introduction

Neurodegenerative diseases like Alzheimer’s disease and Parkinson’s disease are characterized by neuronal loss and chronic neuroinflammation (Amor and Woodroofe, 2014; Nichols et al., 2019). Microglia, being the resident immune cells in the central nervous system (CNS), are the central mediators of neuroinflammation to control host defense and tissue degeneration/regeneration (Wolf et al., 2017). Under neurodegenerative environment, microglia change into a pro-inflammatory phenotype with the activation of the nuclear factor-kappa B (NF-κB)-related signaling pathways and the excessive expression of pro-inflammatory factors such as tumor necrosis factor α (TNF-α) and inducible nitric oxide synthase (iNOS), leading to neuroinflammation (Michels et al., 2015; Thawkar and Kaur, 2019). The depletion of microglia *in vivo* has been reported to halt the propagation of tau, a central protein to many neurodegenerative diseases (Spillantini and Goedert, 2013; Asai et al., 2015). Thus, attenuating the microglia-induced chronic neuroinflammation have emerged as a promising therapeutic strategy to mitigate the progression of neurodegenerative diseases.

Propofol (2, 6-diisopropylphenol) is a short-acting intravenous (IV) anesthetic agent that is widely used not only in operating rooms but also in the intensive care unit (ICU) (Marik, 2004; Hausburg et al., 2020). Propofol acts by potentiating the γ-aminobutyric acid type A (GABA_A_) receptor-mediated inhibitory tone in the CNS (Marik, 2004). Interestingly, our group, together with other labs, have reported that propofol is able to modulate the phenotype of BV2 microglial cells and assert anti-inflammatory effects (Luo et al., 2018; Wu Q. et al., 2018; Zheng et al., 2018; Liu et al., 2019). Similar results were obtained when primary microglia were treated with propofol (Peng et al., 2014). However, the molecular mechanisms of propofol-mediated microglial phenotype transition remain to be elucidated.

The important regulatory functions of miRNAs in microglial activation have been comprehensively investigated (Guo et al., 2019). It is reported that propofol suppresses inflammatory response in lipopolysaccharide (LPS)-activated BV2 cells *via* miR-155 (Zheng et al., 2018). Whether miRNAs are also involved in the propofol-mediated phenotype regulation of primary microglia remains to be an open question. In the present study, we demonstrated the inhibitory effects of propofol on the activation of primary microglia, which confirmed the results obtained from BV2 cells. We screened the miRNA profiles in microglia after treating with LPS and propofol, and observed that propofol reversed the LPS-induced changes in multiple miRNAs including miR-106, miR-124, miR-185, and miR-9. We further demonstrated that miR-106 is the core miRNA that mediated the protective effects of propofol against the inflammatory responses in microglial cells. We then identified Pi3k/Akt signaling as one of the most affected pathways after miR-106b perturbation of function *via* RNA sequencing (RNA-seq) analysis. Lastly, we showed that miR-106b achieve its anti-inflammatory roles *via* down-regulating the activity of Pi3k/Akt signaling.

## Materials and Methods

### Mice and Microglia Culture

Mouse primary microglia were isolated from neonatal mice using an adapted protocol that is originally utilized in human fetal microglial cells isolation (Wu B. et al., 2018). C57 mice were purchased from Shanghai Model Organisms Center. All procedures were conducted according to protocols approved by the Institutional Animal Care and Use Committee of Tongji University School of Medicine. Postnatal day 1 mouse brains were dissected out after removing peripheral blood vessels and digested at 37°C for 30 min in 0.25% trypsin-EDTA (Gibco) supplemented with 0.05% DNase I (Roche). Digestion was stopped by FBS (Invitrogen) and the tissue sediment was centrifuged at 1500 rpm for 5 min at 4°C. After trituration, dissociated cells were cultured in DMEM (Gibco) with 10 ng/mL GM-CSF and 10% FBS, 50 U penicillin and 50 mg/mL streptomycin at 37°C. Culture dishes were coated with 100 μg/mL Poly-D-Lysine (Sigma) and 5 μg/mL Fibronectin (Sigma). The culture medium was replaced every 3 days. Mouse primary microglia in the neonatal brain tissue-derived microglia-astrocytes mixed cultures were induced to detach by shaking. Floating microglia were collected by 1500 rpm centrifugation. Microglia purity was tested by immunostaining with antibodies against Iba-1 (Cat # 019-19741, WAKO; Cat # AB5607, Abcam), Nestin (Cat # NB100-1604, NOVUS), Tuj1 (Cat # T8860, Sigma), Map2 (Cat # MB0078, Bioworld), Gfap (Cat # AB5541, Millipore), Eaat1 (Cat # AB416, Abcam), and O4 (Cat # O7139, Sigma) (Supplementary Figure 1). BV2 microglial cells were purchased from the American Type Culture Collection (ATCC, Rockville, MD, United States). BV2 cells were cultured in DMEM (Gibco) supplemented with 10% FBS, 100 U/mL penicillin, and 100 μg/mL streptomycin at 37°C. LPS was purchased from Sigma (Cat # L2880) and dissolved in PBS. Propofol was purchased from MedChemExpress (Cat # HY-B0649) and dissolved in DMSO. For LPS stimulation, primary microglia and BV2 cells were pre-treated with LPS with the final concentration of 50 and 100 ng/mL, respectively, for 2 h. For propofol treatment, primary microglia and BV2 cells were incubated in determined doses of propofol for 2 days. For all propofol treatment groups, equal volume of DMSO were used as vehicle controls. For pathway analysis, cells were incubated with 5 μM of 740Y-P (Selleck Chemicals, S7865) or 2.5 μM Wortmannin (Selleck Chemicals, S2758) for 2 days.

### miRNA Mimics/Inhibitor and Transfection

The mimics control with scrambled sequence, miR-106a mimics, miR-106b mimics, miR-124 mimics, inhibitor control with scrambled sequence, miR-106b inhibitor, miR-124 inhibitor, miR-22 inhibitor, and miR-29b inhibitor were purchased from GenePharma (GenePharma Co., Ltd., Shanghai, China). Transfection of miRNA mimics/inhibitor or their corresponding control (20 nM) was performed using the HiPerFect Transfection Reagent (Qiagen) according to the manufacturer’s instruction.

### Quantitative Reverse Transcription Polymerase Chain Reaction

Quantitative reverse transcription polymerase chain reaction (qRT-PCR) was performed as previously described (Xia et al., 2019). The messenger RNA (mRNA) and miRNA were isolated from cells using RNeasy mini kit (Qiagen) according to the manufacturer’s instructions. Genomic DNA was removed using DNase I digestion kit (Qiagen). cDNA was synthesized using miScript II RT kit (Qiagen). Transcripts were amplified using specific primer sets (Supplementary Table 1) and SYBR green PCR kit (Qiagen) with the ABI7500 (Applied Biosystems). Reactions were run in triplicates for each sample and no-template blanks were used as negative controls. Values were normalized to the *Gapdh* (for mRNA) and U6 snRNA (for miRNA).

### Western Blotting

Western blotting was performed as previously described (Gao et al., 2019). Cells were lysed in RIPA lysis and extraction buffer (ThermoFisher) containing a protease inhibitor cocktail (Sigma). Protein concentrations were determined with a BCA Protein Assay Kit (Pierce). Proteins (20–30 mg) from cell lysates were separated using sodium dodecyl sulfate polyacrylamide gel electrophoresis (SDS-PAGE) and electrophoretic transferred to polyvinylidene fluoride membranes (Millipore and Bio-Rad). Membranes were incubated with primary antibodies for β-actin (rabbit, Cell Signaling Technology, 1:1000), Akt (rabbit, Cell Signaling Technology, 1:2000), phospho-Akt (rabbit, Cell Signaling Technology, 1:2000), overnight at 4°C followed by a secondary anti-rabbit or anti-mouse antibody (Cell Signaling Technologies, 1:10,000) incubation. Pierce ECL Western Blotting Substrate (ThermoFisher) was used for visualization of the protein bands. CanoScan 9950F scanner was used to scan the membrane and ImageJ program was used to analyze the acquired images.

### RNA Sequencing and Bioinformatics Analysis

RNA sample processing was performed using the Illumina HiSeq platform. Sequencing libraries were generated using NEBNext^®^ Ultra^TM^ RNA Library Prep Kit for Illumina^®^ following manufacturer’s instructions and index codes were added to attribute sequences to each sample. The clustering of the index-coded samples was performed on a cBot Cluster Generation System using TruSeq PE Cluster Kit v3-cBot-HS following manufacturer’s instructions. The prepared libraries were sequenced and 125/150 bp paired-end reads were generated. Index of the reference genome was built paired-end clean reads were aligned to the reference genome using Hisat2 v2.0.5. RNA-seq reads counting was done using featureCounts v1.5.0-p3. Fragments per kilobase of transcript per million fragments mapped (FPKM) of each gene was determined based on the length of the gene and reads count mapped to this gene. Differential expression analysis was performed using the DESeq2 R package. *p*-Values and *q*-values were adjusted using the Benjamini and Hochberg’s approach for controlling the false discovery rate. Genes with *q*-value < 0.05 were considered as differentially expressed. Differentially expressed genes (DEGs) with over 1.5-fold changes were mapped to Gene Ontology (GO) and Kyoto Encyclopedia of Genes and Genomes (KEGG) pathways analysis. GO and KEGG enrichment analysis was performed using the Database for Annotation, Visualization and Integrated Discovery (DAVID).^1^

### Statistical Analyses

All results are presented as the means of at least three independent experiments ± SD. Data from two groups were evaluated statistically by two-tailed, paired or unpaired Student *t*-test, and that among more than two groups were assessed with the parametric one-way ANOVA with *post hoc* Bonferroni test. Significance was considered when *p* < 0.05.

## Results

### Propofol Attenuates LPS-Stimulated Microglial Activation

In primary mouse microglia, 2 h LPS pre-treatment significantly increased the expression levels of transcripts corresponding to *Tnf* and *Nos2* (Supplementary Figure 2A). The up-regulation of transcript expression levels of NF-κB pathway components including *Ticam1*, *Myd88*, *Irf3*, and *Nfkb1* has also been observed, confirming the activation of microglia exposed to LPS (Supplementary Figure 2B). We then treated microglia with propofol in dose gradient from 10, 20, 50, to 100 μM. The results showed that only 100 μM propofol significantly increased the proportions of apoptotic cells under both normal (Supplementary Figure 3) and LPS-exposed (Supplementary Figure 4) conditions. TUNEL results were confirmed by CCK8 assay (Supplementary Figure 5). Thus, high dose of propofol (100 μM) exhibited strong cytotoxicity and was excluded from following studies.

**FIGURE 1:**
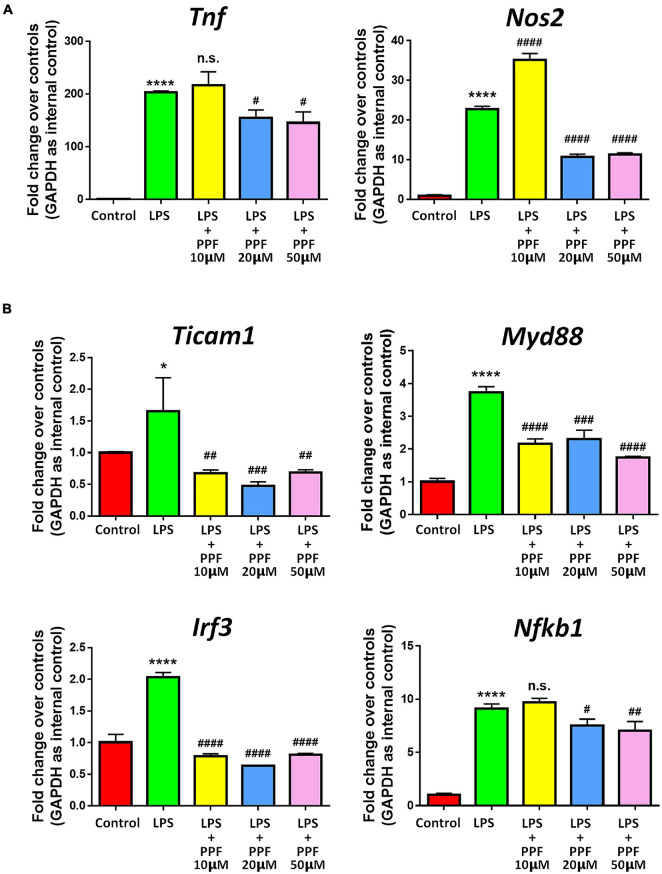
Propofol inhibits LPS-stimulated microglia. Primary mouse microglia were stimulated by LPS, followed by propofol treatment for 2 days. **(A)** The transcript levels of pro-inflammatory genes *Tnf* and *Nos2* in microglia were determined by qRT-PCR. **(B)** The transcript levels of NF-κB signaling component genes *Ticam1*, *Myd88*, *Irf3*, and *Nfkb1* in microglia were determined by qRT-PCR. Data were represented as mean ± SD from three independent experiments. The symbols * and **** denote *p* < 0.05 and *p* < 0.0001, respectively, in comparison with control group. The symbols ^#^, ^##^, ^###^, and ^####^ denote *p* < 0.05, *p* < 0.01, *p* < 0.001, and *p* < 0.0001, respectively, in comparison with LPS group. n.s. denotes non-significant. PPF, propofol.

To investigate the effects of propofol on microglial activation, we examined the expression of transcripts corresponding to microglial activation-related genes after treating microglia with different dose of propofol for 2 days. qRT-PCR results revealed that propofol attenuated LPS-induced expression of *Tnf* and *Nos2* transcripts in microglia in a concentration-dependent manner (Figure 1A). The expression of *Ticam1*, *Myd88*, *Irf3*, and *Nfkb1* transcripts were also reduced with the increase of propofol concentration (Figure 1B). We then cultured LPS-stimulated microglia with propofol for 12, 24, 48, 96 h to determine the time-point for following studies. The qRT-PCR results indicated that propofol exhibited stable anti-inflammatory effects at 48 h (Supplementary Figure 6). Therefore, 2 days 50 μM propofol treatment was used in following experiment. The qRT-PCR results were corroborated by western blotting that propofol treatment reversed the LPS-induced excessive pro-inflammatory protein CD86 expression (Supplementary Figure 7). Thus, our results demonstrated the anti-inflammatory role of propofol on microglial activation.

### Propofol Alters miRNA Profile in Activated Microglia

**FIGURE 2:**
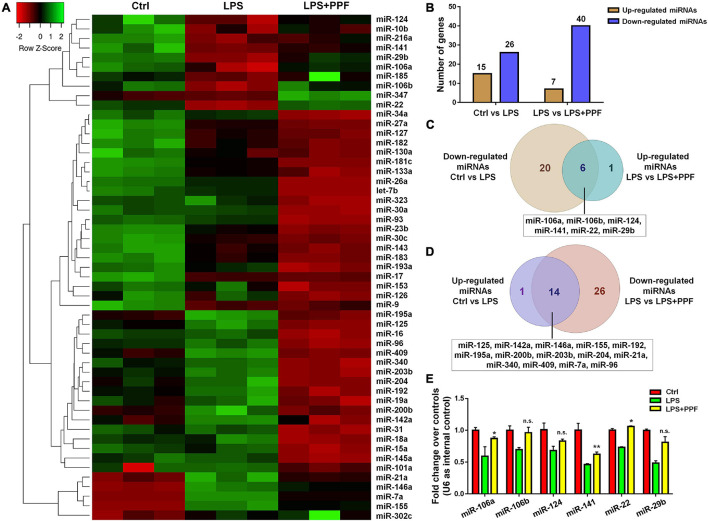
APP-EXO promote APP expression in recipient cells. **(A)** Hierarchical cluster analysis of 53 miRNAs expressed in microglia with LPS and propofol treatment. **(B)** The numbers of up- and down-regulated miRNAs in comparison between either LPS and control groups or LPS + PPF and LPS groups. **(C)** Venn diagram represents the numbers of down-regulated (light brown) miRNAs in LPS group in comparison with control group and up-regulated (light blue) miRNAs in LPS + PPF group in comparison with LPS group. **(D)** Venn diagram represents the numbers of up-regulated (lavender) miRNAs in LPS group in comparison with control group and down-regulated (light pink) miRNAs in LPS + PPF group in comparison with LPS group. **(E)** The expression levels of miR-106a, miR-106b, miR-124, miR-141, miR-22, and miR-29b in microglia with LPS and propofol treatment were determined by qRT-PCR. Data were represented as mean ± SD from three independent experiments. The symbols * and ** denote *p* < 0.05 and *p* < 0.01, respectively, in comparison with control group. n.s. denotes non-significant.

To elucidate the involvement of miRNAs in propofol-mediated microglial phenotype regulation, we determined the expression profiles of 55 highly conserved miRNAs in LPS or/and propofol treated microglia. All miRNAs were detected by qRT-PCR analysis except for miR-144 and miR-25 (Figure 2A). Among all detected miRNAs, 15 and 26 of them were up-regulated and down-regulated, respectively, in LPS-treated microglia, compared with controls (Figure 2B). Besides, 7 and 40 miRNAs were up-regulated and down-regulated, respectively, in microglia incubated with both LPS and propofol, compared with LPS-treated ones (Figure 2B). Moreover, among 26 down-regulated miRNAs in LPS-treated microglia, 6 of them (miR-106a, miR-106b, miR-124, miR-141, miR-22, and miR-29b) were up-regulated after being exposed to propofol (Figure 2C). In contrast, among 15 up-regulated miRNAs in LPS-treated microglia, 14 of them (miR-125, miR-142a, miR-146a, miR-155, miR-192, miR-195a, miR-200b, miR-203b, miR-204, miR-21a, miR-340, miR-409, miR-7a, and miR-96) were down-regulated after being exposed to propofol (Figure 2D). Therefore, our results indicated that propofol altered the miRNA profile in LPS-activated microglia.

### miR-106b Mediates the Anti-inflammatory Effects of Propofol

We next investigated the involvement of miRNA candidates in propofol-attenuated microglial activation. Since literatures have reported propofol inhibited microglial activation *via* suppressing miR-155 expression (Zheng et al., 2018), we focused on the 6 miRNAs exhibiting opposite expression patterns of miR-155 in the study (Figures 2C,D). The qRT-PCR results suggested that propofol brought the reduced expression levels of miR-106b, miR-124, miR-22, and miR-29b, after LPS treatment, back to the levels comparable to or even higher than that of controls (Figure 2E).

**FIGURE 3:**
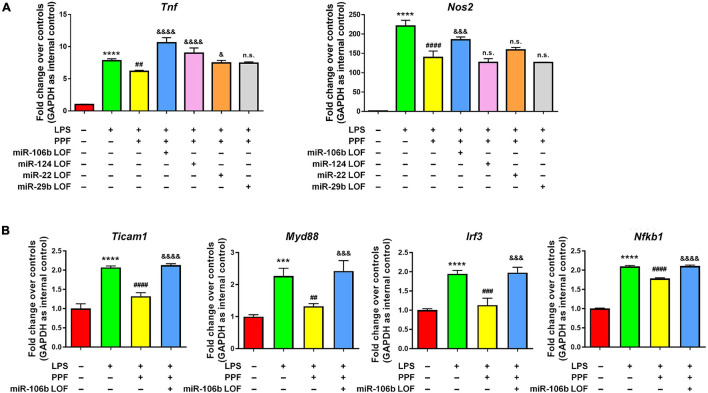
miR-106b mediates the anti-inflammatory effects of propofol. Primary mouse microglia were stimulated by LPS, followed by propofol treatment and the transfection of either miR-106b, miR-124, miR-22, or miR-29b inhibitors for 2 days. **(A)** The transcript levels of pro-inflammatory genes *Tnf* and *Nos2* in microglia were determined by qRT-PCR. **(B)** The transcript levels of NF-κB signaling component genes *Ticam1*, *Myd88*, *Irf3*, and *Nfkb1* in microglia were determined by qRT-PCR. Data were represented as mean ± SD from three independent experiments. The symbols *** and **** denote *p* < 0.001 and *p* < 0.0001, respectively, in comparison with control group. The symbols ^##^, ^###^, and ^####^ denote *p* < 0.01, *p* < 0.001, and *p* < 0.0001, respectively, in comparison with LPS group. The symbols ^&^, ^&&&^, and ^&&&&^ denote *p* < 0.05, *p* < 0.001, and *p* < 0.0001, respectively, in comparison with LPS + PPF group. PPF, propofol. n.s. denotes non-significant.

To understand whether aforementioned four miRNAs mediated the anti-inflammatory effects of propofol, we carried out miRNA loss-of-function (LOF) approach *via* transfecting propofol-treated microglia with either miR-106b, miR-124, miR-22, and miR-29b inhibitors, or inhibitor controls for 2 days. qRT-PCR results revealed that the knockdown of miR-106b reversed the propofol-induced *Tnf* and *Nos2* expression inhibition (Figure 3A). The knockdown of miR-124 only abrogated the inhibitory effects of propofol on *Tnf* expression, but not *Nos2* expression. In addition, miR-22 and miR-29b LOF had no effects on *Tnf* and *Nos2* expression. These results implied miR-106b as a key down-stream factor of propofol in the regulation of microglial activation. To confirm our finding, we also examined the expression levels of *Ticam1*, *Myd88*, *Irf3*, and *Nfkb1* transcripts after miR-106b LOF (Figure 3B). The knockdown of miR-106b expression erased the inhibitory effects of propofol on the expression of transcripts corresponding to NF-κB pathway components.

**FIGURE 4:**
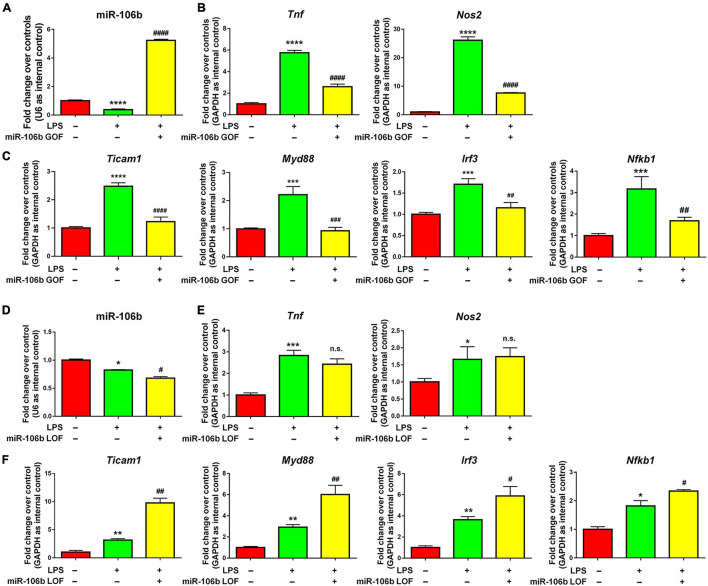
miR-106b suppresses LPS-stimulated microglia. **(A)** Primary mouse microglia were stimulated by LPS, followed by the transfection of miR-106b mimics and mimics control for 2 days. miR-106b GOF in microglia was validated by qRT-PCR. **(B)** The transcript levels of pro-inflammatory genes *Tnf* and *Nos2* in microglia were determined by qRT-PCR. **(C)** The transcript levels of NF-κB signaling component genes *Ticam1*, *Myd88*, *Irf3*, and *Nfkb1* in microglia were determined by qRT-PCR. **(D)** Primary mouse microglia were stimulated by LPS, followed by the transfection of miR-106b mimics and mimics control for 2 days. miR-106b LOF in microglia was validated by qRT-PCR. **(E)** The transcript levels of pro-inflammatory genes *Tnf* and *Nos2* in microglia were determined by qRT-PCR. **(F)** The transcript levels of NF-κB signaling component genes *Ticam1*, *Myd88*, *Irf3*, and *Nfkb1* in microglia were determined by qRT-PCR. Data were represented as mean ± SD from three independent experiments. The symbols *, **, *** and **** denote *p* < 0.05, *p* < 0.01, *p* < 0.001 and *p* < 0.0001, respectively, in comparison with control group. The symbols ^#^, ^##^, ^###^, and ^####^ denote *p* < 0.05, *p* < 0.01, *p* < 0.001, and *p* < 0.0001, respectively, in comparison with LPS group. n.s. denotes non-significant.

The further validate the anti-inflammatory roles of miR-106b, we performed miR-106b gain-of-function (GOF) approach *via* transfecting LPS-treated microglia with either miR-106b mimics or mimics controls for 2 days. The transfection efficiency was been corroborated by determining the miR-106b expression levels *via* qRT-PCR (Figure 4A). qRT-PCR results further revealed that miR-106b GOF reversed the up-regulated expression of transcripts corresponding to *Tnf*, *Nos2* (Figure 4B) and NF-κB pathway component genes (Figure 4C). The effects of miR-106b GOF in the presence of propofol were also examined. The overexpression of miR-106b could further reduce the expression of *Tnf* in propofol-treated LPS-stimulated microglia (Supplementary Figure 8). Furthermore, the miR-106b LOF has been carried out *via* transfecting LPS-stimulated microglia with either miR-106b inhibitor or inhibitor controls for 2 days. The miR-106b knockdown efficiency was been confirmed *via* qRT-PCR (Figure 4D). qRT-PCR results that miR-106b LOF significantly elevated the expression levels of NF-κB pathway component genes (Figure 4F). However, we did not observe significant difference of *Tnf* and *Nos2* expression after knocking down miR-106b (Figure 4E). It could be explained that miR-106b LOF in LPS-induced miR-106b inhibition condition might not be as effective as miR-106b GOF. Taken together, our results suggested that propofol functions as a microglial activation suppressor through anti-inflammatory miRNA miR-106b.

### miR-106 Inhibits LPS-Stimulated Microglial Activation *via* Pi3k/Akt Pathway

**FIGURE 5:**
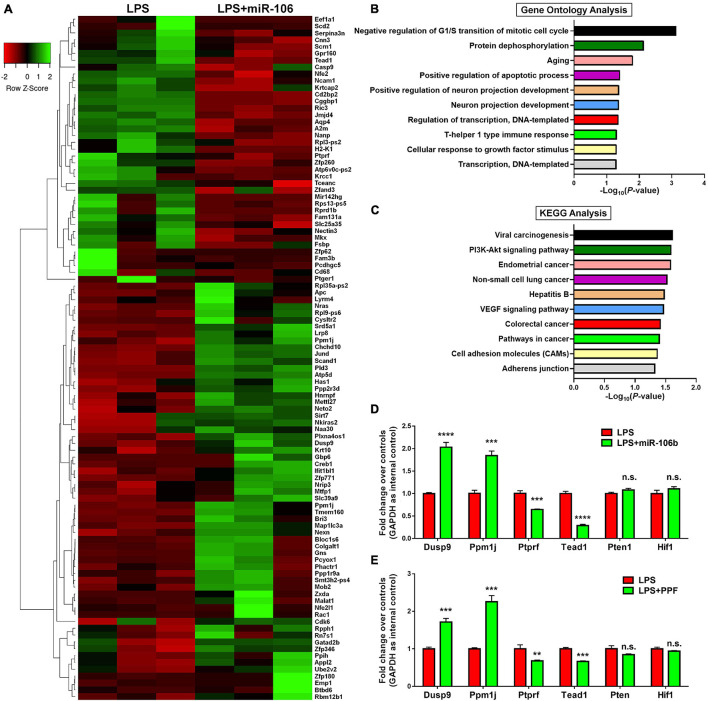
miR-106b significantly alters the gene expression in LPS-stimulated microglia. **(A)** The heatmap and hierarchical cluster analysis of top 100 genes with the highest fold changes between LPS and LPS + miR-106b GOF groups, identified by RNA-seq assay. **(B)** The Gene Ontology (GO) analysis of differentially expressed genes between LPS and LPS + miR-106b GOF groups. **(C)** The Kyoto Encyclopedia of Genes and Genomes (KEGG) analysis of differentially expressed genes between LPS and LPS + miR-106b GOF groups. **(D)** Independent qRT-PCR analyses of up-regulated (*Dusp9* and *Ppm1j*), down-regulated (*Ptprf* and *Tead1*), and unchanged genes (*Pten* and *Hif1*) identified by RNA-seq assay. **(E)** The transcript levels of RNA-seq assay-identified up-regulated (*Dusp9* and *Ppm1j*), down-regulated (*Ptprf* and *Tead1*), and unchanged genes (*Pten* and *Hif1*) in propofol-treated microglia were determined by qRT-PCR. Data were represented as mean ± SD from three independent experiments. The symbols **, ***, and **** denote *p* < 0.01, *p* < 0.001, and *p* < 0.0001, respectively. GOF, gain-of-function. n.s. denotes non-significant.

To understand the potential downstream effectors of miR-106b in the regulation of microglial activation, we examined the global gene expression profiles of miR-106b GOF microglia under LPS stimulation. The RNA-seq results revealed that 43 genes were up-regulated and 91 genes were down-regulated in LPS + miR-106b group, compared with LPS group (Figure 5A). The RNA-seq analysis identified the overexpression of miR-106b down-regulated pro-inflammatory genes including *Casp9* (Chong et al., 2004), *Rac1* (D’Ambrosi et al., 2014), *Ptger1* (Carlson et al., 2009), *Il6st* (El Husseini et al., 2018), *Fam3b* (Peng et al., 2016), and up-regulated anti-inflammatory genes such as *Nkiras2* (Luoreng et al., 2021), which further confirmed our finding (Figure 5A). GO analysis revealed that the DEGs were enriched in “Negative regulation of G1/S transition of mitotic cell cycle,” “Protein dephosphorylation,” and “Aging” terms, which might be related to key aspects of microglial activation including reactive gliosis, inflammatory signaling regulation, and age-induced microglial dysfunction (Figure 5B). KEGG analysis suggested that the DEGs were also enriched in pathways that are strongly associated with microglial activation including Pi3k/Akt and VEGF signaling pathways (Figure 5C). We than randomly selected 2 up-regulated (*Dusp9* and *Ppm1j*), down-regulated (*Ptprf* and *Tead1*), and unchanged genes (*Pten* and *Hif1*) and independently confirmed the RNA-seq data by qRT-PCR (Figure 5D). We next examined that whether the expression levels of aforementioned genes were similarly regulated when microglia were exposed to propofol. The qRT-PCR results revealed that the expression patterns of *Dusp9*, *Ppm1j*, *Ptprf*, *Tead1*, *Pten*, and, *Hif1* transcripts in propofol-exposed microglia were consistent with that of miR-106b GOF group, when compared with LPS groups, further confirming miR-106b as the key downstream effector of propofol (Figure 5E).

**FIGURE 6:**
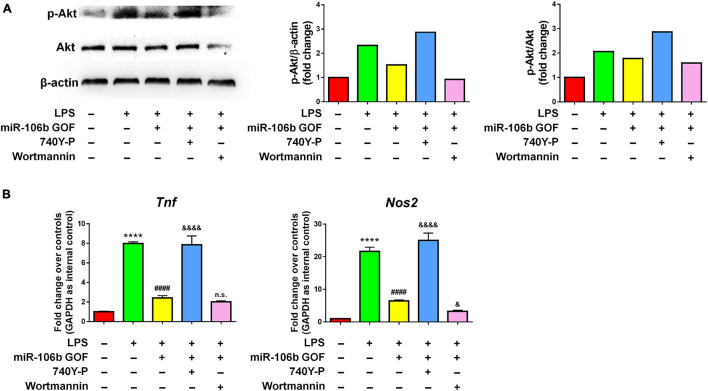
The inhibitory effects of miR-106b on microglial activation are mediated by Pi3k/Akt pathway. Primary mouse microglia were stimulated by LPS, followed by the transfection of miR-106b mimics and the treatment of either 740Y-P or Wortmannin for 2 days. **(A)** The representative western blots showing the expression of p-Akt and Akt in (left). Densitometric quantifications were presented on the right. **(B)** The transcript levels of pro-inflammatory genes *Tnf* and *Nos2* in microglia were determined by qRT-PCR. Data were represented as mean ± SD from three independent experiments. The symbol **** denotes *p* < 0.0001 in comparison with control group. The symbol ^####^ denotes *p* < 0.0001 in comparison with LPS group. The symbols ^&^ and ^&&&&^ denote *p* < 0.05 and *p* < 0.0001, respectively, in comparison with LPS + miR-106b GOF group. GOF, gain-of-function. n.s. denotes non-significant.

Since the RNA-seq analysis implied Pi3k/Akt signaling pathway as one of the most regulated in miR-106b GOF microglia, we firstly confirmed the RNA-seq data by western blotting. The results revealed that LPS significantly enhanced the levels of p-Akt, which was abolished by miR-106b GOF, matching with RNA-seq analysis (Figure 6A). More importantly, propofol treatment also reversed the hyperphosphorylation of Akt post LPS stimulation (Supplementary Figure 9), suggesting Pi3k/Akt signaling as downstream pathway of propofol-miR-106b axis. We then investigated the roles of Pi3k/Akt signaling pathway in the miR-106b-mediated inhibition of microglial activation using Pi3k/Akt pathway specific agonist 740Y-P and antagonist Wortmannin. The western blotting results suggested the levels of p-Akt were significantly up-regulated and down-regulated when cells were treated with 740Y-P and Wortmannin, respectively, confirming the 740Y-P-induced and Wortmannin-inhibited Pi3k/Akt pathway activation (Figure 6A). Importantly, the treatment of 740Y-P abrogated the miR-106b-mediated inhibition of *TNF* and *Nos2* expression that was induced by LPS stimulation (Figure 6B). In contrast, the treatment of Wortmannin further reduced the *Nos2* expression levels, compared with miR-106 GOF group (Figure 6B). These observations suggest Pi3k/Akt pathway as an important downstream signaling of miR-106b in the regulation of microglial activation.

## Discussion

Propofol, as one of the most commonly used IV drugs for the anesthesia induction and maintenance during surgical procedures and critical care sedation in ICUs, has been reported to have potential therapeutic effects on various neurological disorders including neurodegenerative diseases (Shao et al., 2014), stroke (Hausburg et al., 2020), and depression (Mickey et al., 2018). Here, our results, together with our previous study (Liu et al., 2019), have demonstrated the anti-inflammatory roles of propofol in both microglial cell line and primary microglia. Our findings match with the results obtained from various *in vitro* and *in vivo* studies, implying propofol as a promising drug candidate in treating neuroinflammation and its related diseases (Peng et al., 2014; Liu et al., 2017; Luo et al., 2018; Wu Q. et al., 2018; Zheng et al., 2018). It is worth-noting that the anti-inflammatory effects of propofol were highly dose-dependent. We have demonstrated that high dose of propofol induces severe microglial cell death (Supplementary Figures 3, 4). Animal study further showed that the administration of high dose of propofol during brain development causes neonatal brain cell death and neurodegeneration, resulting in functional deficits in adulthood (Fredriksson et al., 2007). Thus, it is important to determine the working dose of propofol before any potential clinical applications.

The mechanisms for propofol-mediated microglial phenotype transition are poorly understood. In this study, we identified miR-106b as a key downstream factor of propofol. To date, the involvement of miR-106b in microglial activation remains to be evidenced, however, other miR-17 family members that share the same seed sequence with miR-106b (miR-17, miR-106a, miR-106b, miR-20a, miR-20b, and miR-93) have been reported to regulate inflammatory responses of microglia. Jadhav et al. (2014) and Gamdzyk et al. (2020) both reported that miR-17 inhibits microglial activation *via* suppressing reactive oxygen species (ROS) producer NOXs expression and NLRP3 activation. Similarly, miR-93 has been found to inhibit inflammatory responses of microglia through directly targeting *STAT3* and interleukin-1 receptor-associated kinase *IRAK4* (Tian et al., 2017; Wang et al., 2021). Notably, in a vascular dementia rat model, miR-93 has been reported to enhance microglial activation that contributes to the exacerbation of cognitive impairment (Wang L. et al., 2020). The conflicting observations imply the complexity of miR-17 family-mediated microglia regulation and the importance to investigate each individual miRNA in this family. We found that the reduction of miR-106b expression levels in activated microglia significantly suppresses the expression of pro-inflammatory factors and NF-κB components. Our study is the first one that demonstrates the inhibitory roles of miR-106 in microglial activation, filling the knowledge gap in the miR-17 family-mediated neuroinflammatory regulation. It is worth-noting that miR-106a showed similar expression pattern to miR-106b after treating with LPS and propofol (Figure 2E). The GOF approach revealed that miR-106a functioned as an anti-inflammatory miRNA as well (Supplementary Figure 10). However, since propofol did not exhibit the same promoting effects on the expression of miR-106a versus miR-106b, whether miR-106a also acts as a downstream factor of propofol remains to be examined.

Interestingly, although we did not find that the miR-124 LOF abolished the inhibitory effects of propofol on *Nos2* expression, miR-124 LOF did elevate *TNF* expression levels, implying a potential role of miR-124 in microglial activation regulation (Figure 3A). Various literatures have indicated miR-124 as a potent anti-inflammatory agent which affects microglia after brain injury including intracerebral hemorrhage and surgical trauma (Yu et al., 2017; Chen et al., 2019). Similar roles of miR-124 have also been found in animal models of neurodegenerative diseases (Yao et al., 2018) and depression (Lou et al., 2019). However, it is also reported that miR-124 activates microglia and increases the expression levels of pro-inflammatory cytokines including *Il1b*, *Tnf*, and *Il6 in vivo*, implicating dual and opposing roles of miR-124 in neuroinflammation (Brennan et al., 2016). In our study, we also performed miR-124 GOF in LPS-stimulated microglia. The results suggest that miR-124 did not reverse the LPS-induced *Tnf* overexpression, and caused a slight but significant reduction of *Nos2* expression (Supplementary Figure 11). Thus, our *in vitro* study revealed that, compared with miR-106, miR-124 might play an inconsequential or a relatively minor role in microglial activation, which needs to be further confirmed *in vivo*.

Another key finding of this study is that we identified Pi3k/Akt pathway as a downstream signaling of miR-106 in microglial activation regulation. To date, conflicting results have been obtained in the perspective of Pi3k/Akt pathway-mediated microglia regulation. Saponaro et al. (2012) have reported that this signaling cascade was activated by many cytokines and LPS in microglia. The inhibition of Pi3k/Akt pathway by curcumin suppresses LPS-stimulated microglial activation (Cianciulli et al., 2016). However, opposite trends have been observed by Wang et al. (2015) that LPS inhibits the phosphorylation of Akt in LPS-challenged microglia. The activation of Pi3k/Akt pathway suppresses the pro-inflammatory phenotype of microglia, ameliorating neuroinflammatory response and cognitive impairment in AD mice (Wang Y. et al., 2020). In our study, our results reveal Pi3k/Akt pathway as a pro-inflammatory signaling in defined conditions, indicating the regulation of the Pi3k/Akt axis as a possible approach to develop new treatments for neurodegenerative diseases by inhibiting microglial activation. Moreover, although we have shown the contribution of Pi3k/Akt pathway in miR-106b-mediated microglial activation modulation, we did not identify the direct target of miR-106b. Surprisingly, none of DEGs identified by RNA-seq has complementary sites for miR-106b. One possibility is that miR-106b achieve its function not through classic miRNA-mRNA 3′ UTR complementary base pairing. For instance, miRNAs may act as a chaperone to modify mRNA structure and facilitate ribosome access to mRNA by binding to the 5′ UTR upstream of the internal ribosome entry site (Jopling et al., 2005). Besides, certain miRNAs that are prominently localized in the nucleus have been associated with transcriptional regulation *via* binding to the promoter regions in sense and antisense orientations (Di Mauro et al., 2019; Stavast and Erkeland, 2019; Younger et al., 2009). More comprehensive investigations are required to understand whether miR-106b regulates microglial activation through these mechanisms.

## Conclusion

In summary, our study has demonstrated that propofol significantly inhibit LPS-stimulated microglial activation *in vitro*. Through high-throughput screening and comprehensive function verification experiments, we have further identified miR-106b/Pi3k/Akt axis as a key downstream signaling of propofol-mediated anti-inflammatory responses. Thus, our study provides a possible mechanism for the inhibitory effects of propofol on microglial activation, implying propofol as a potential therapeutic reagent for the treatment of neuroinflammation-related neurodegenerative diseases and other neurological disorders.

## Data Availability Statement

The datasets presented in this study can be found in online repositories. The names of the repository/repositories and accession number(s) can be found below: ArrayExpress, E-MTAB-10946.

## Ethics Statement

The animal study was reviewed and approved by the Institutional Animal Care and Use Committee of Tongji University School of Medicine.

## Author Contributions

JL, XX, and JZ conceptualized the project and designed the experiments. JL, PA, YS, XY, CL, YL, and XX performed the experiments. JL, PA, YS, XY, and XX analyzed the data. XX prepared the manuscript. All authors read and approved the final manuscript.

## Conflict of Interest

The authors declare that the research was conducted in the absence of any commercial or financial relationships that could be construed as a potential conflict of interest.

## Publisher’s Note

All claims expressed in this article are solely those of the authors and do not necessarily represent those of their affiliated organizations, or those of the publisher, the editors and the reviewers. Any product that may be evaluated in this article, or claim that may be made by its manufacturer, is not guaranteed or endorsed by the publisher.

## Footnotes

1
http://david.ncifcrf.gov/
