# Supplementary material for: Propofol Inhibits Microglial Activation via miR-106b/Pi3k/Akt Axis
Source: Front Cell Neurosci. 2021 Oct 28;15:768364. doi: 10.3389/fncel.2021.768364 (PMC8581742; doi:10.3389/fncel.2021.768364)
Supplement: Supplementary file 10 [file Data_Sheet_1.docx]

**Supplementary Information**

**Propofol inhibits microglial activation via miR-106b/Pi3k/Akt axis**

**Jianhui Liu, Yiyan Sun, Pu Ai, Xiaoyu Yang, Chunhong Li, Yi Wang, Yihan Liu, Xiaohuan Xia, Jialin C. Zheng**

**Supplementary Outline**

Supplementary Figure 1

Supplementary Figure 2

Supplementary Figure 3

Supplementary Figure 4

Supplementary Figure 5

Supplementary Figure 6

Supplementary Figure 7

Supplementary Figure 8

Supplementary Figure 9

Supplementary Figure 10

Supplementary Figure 11

Supplementary Table 1

Supplementary Materials and Methods


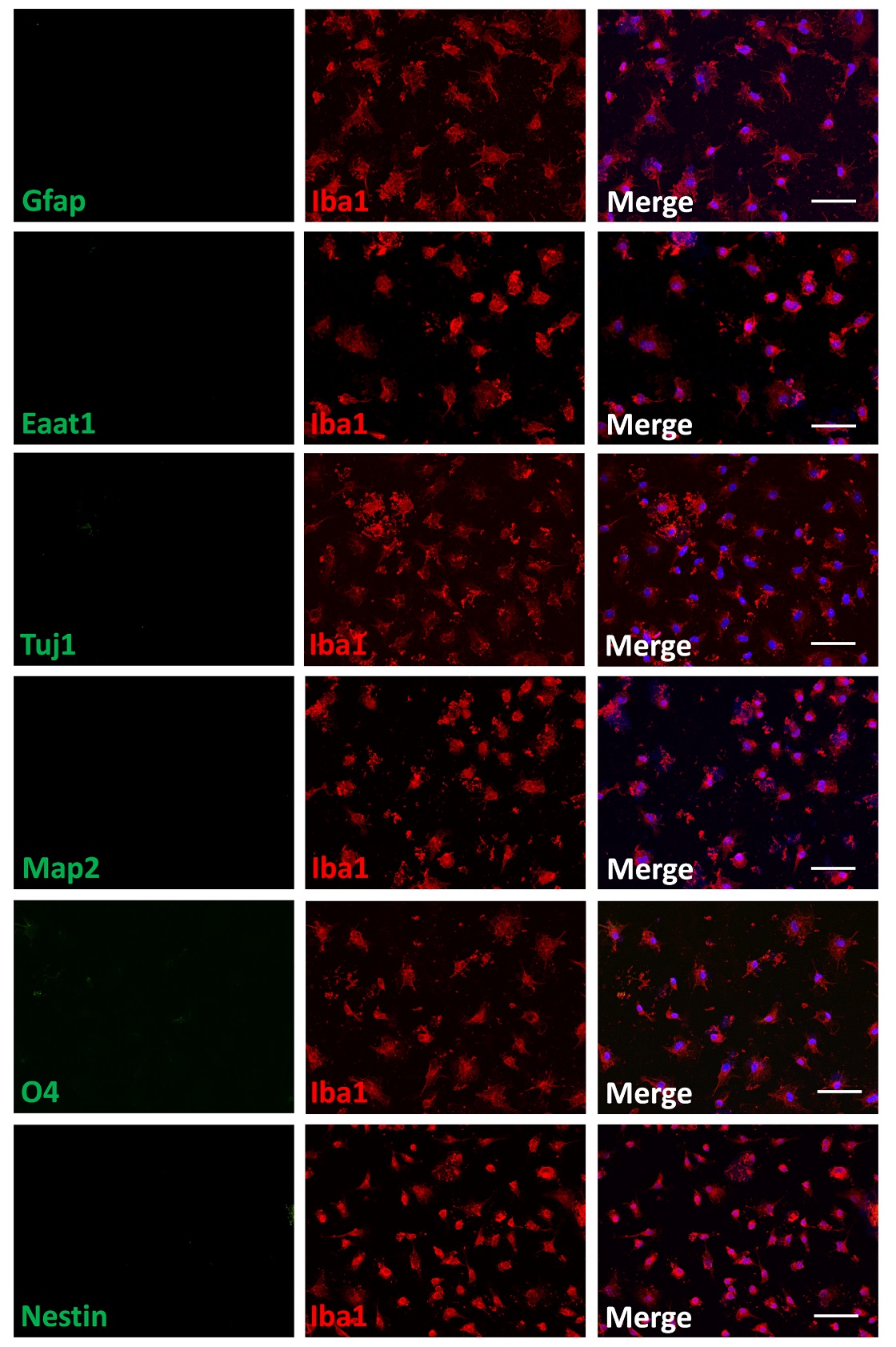


**Supplementary Figure 1. Microglia enrichment validation**.

Mouse primary microglia were counterstained with Iba1 (microglia marker) and either neuronal markers (Tuj1/Map2), astroglial markers (Gfap/Eaat1), oligodendroglial markers (O4), or neural stem cell marker (Nestin) for the validation of the purity of microglia culture. Scale bar: 50 μm.

**
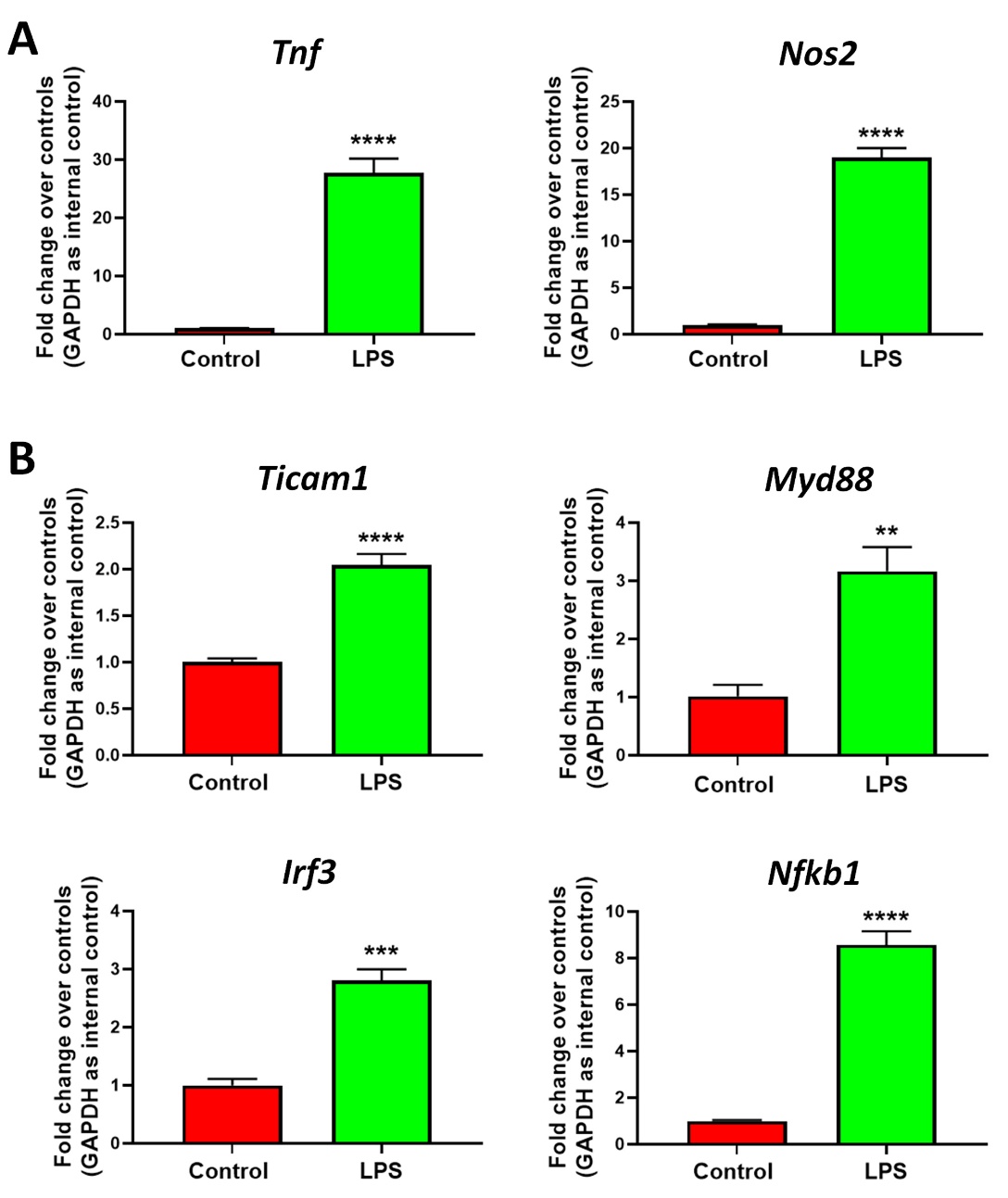
**

**Supplementary Figure 2. LPS stimulates microglial activation**.

(**A**) Microglia were treated with either PBS control or LPS for 2 hours and collected at 2 days. The expression levels of *Tnf* and *Nos2* transcripts were determined by RT-qPCR. (**B**) The expression levels of *Ticam1*, *Myd88*, *Irf3*, and *Nfkb1* transcripts were determined by RT-qPCR. Data were represented as mean±s.d. from three independent experiments. **, ***, and **** denote *p* < 0.01, *p* < 0.001 and *p* < 0.0001, respectively.


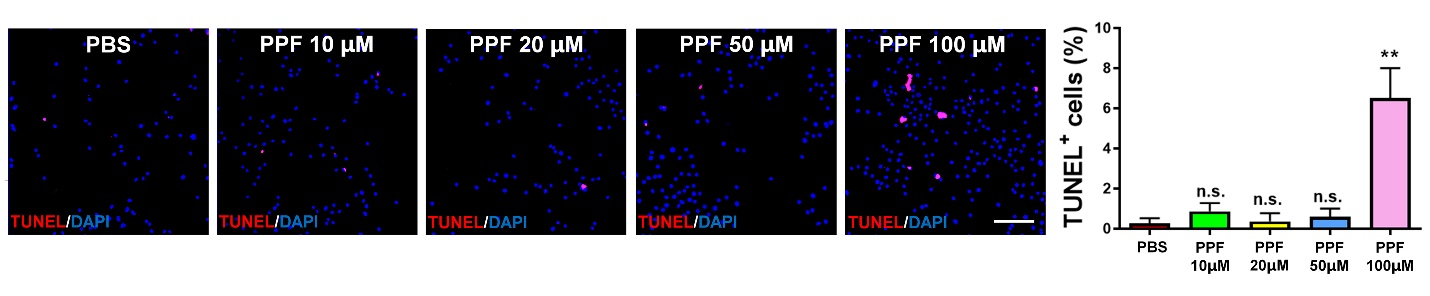


**Supplementary Figure 3. The cytotoxicity of propofol on primary microglia**.

The cytotoxicity of 2 days treatment of propofol on primary microglia were determined by TUNEL assay. Data were represented as mean±s.d. from three independent experiments. ** denotes *p* < 0.01. Scale bar: 100 μm. PPF: propofol.


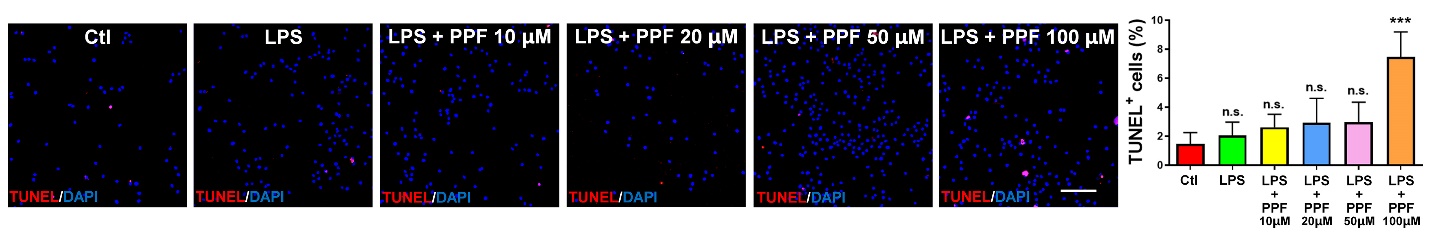


**Supplementary Figure 4. The cytotoxicity of propofol on LPS-stimulated microglia**.

The cytotoxicity of 2 days treatment propofol on LPS-stimulated primary microglia were determined by TUNEL assay. Data were represented as mean±s.d. from three independent experiments. *** denotes *p* < 0.001. Scale bar: 100 μm. PPF: propofol.


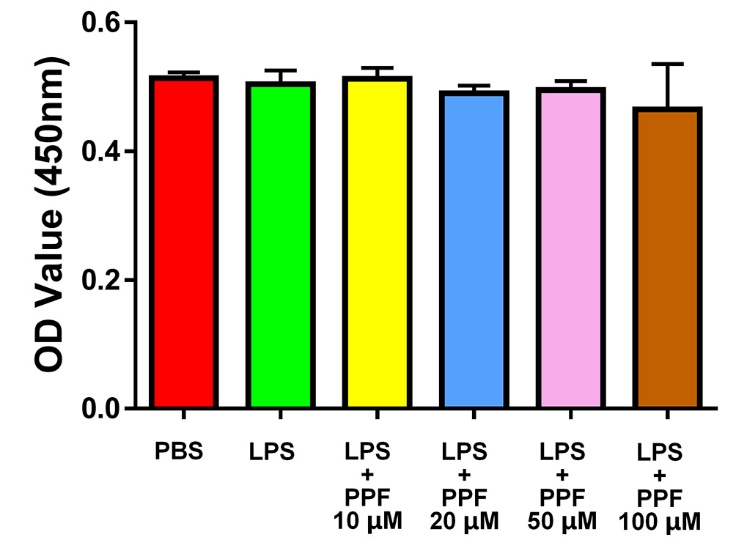


**Supplementary Figure 5. The viability of propofol-treated microglia**.

The viability of propofol-treated LPS-stimulated microglia were determined by CCK8. Data were represented as mean±s.d. from three independent experiments. PPF: propofol.


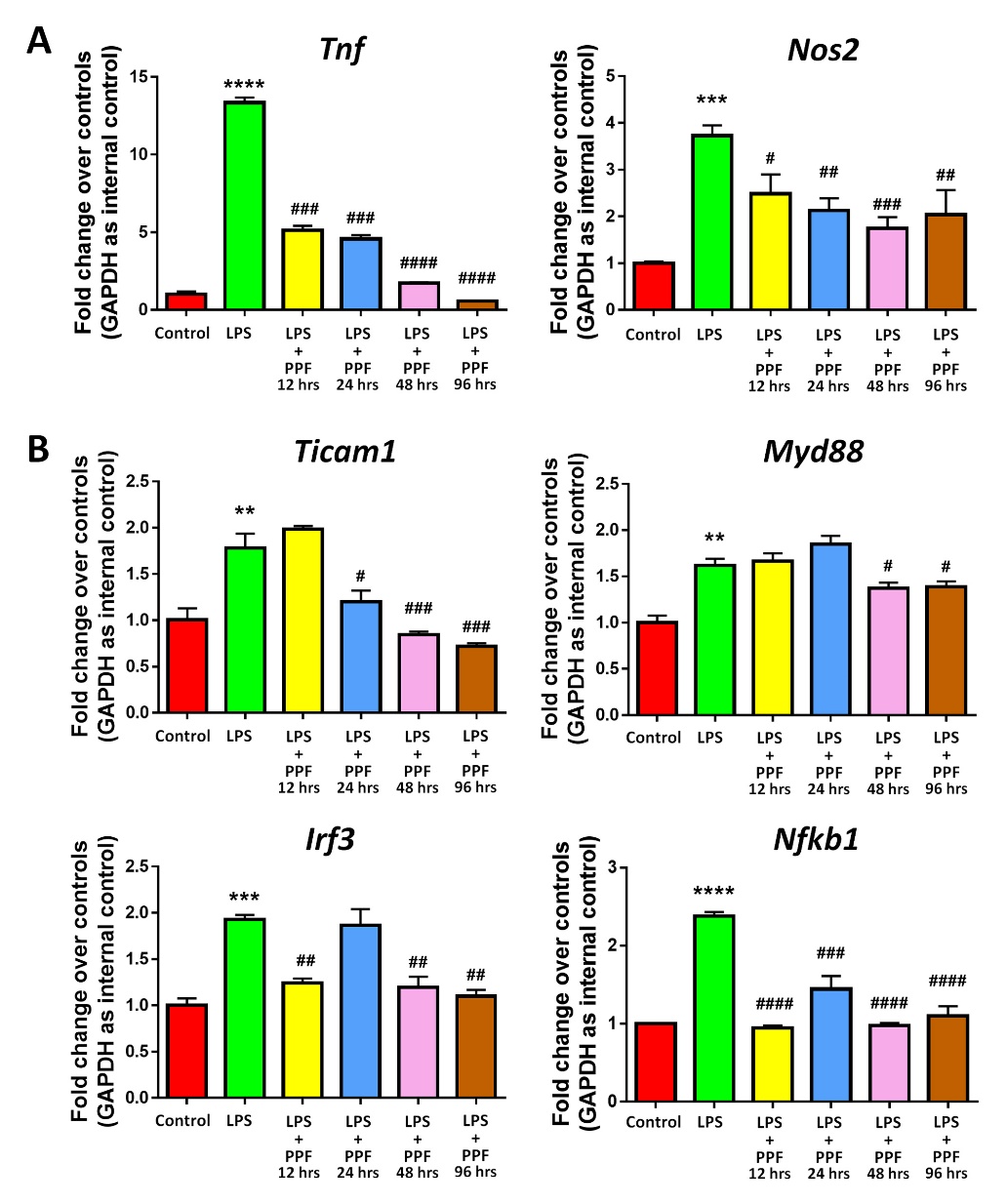


**Supplementary Figure 6. The effects of propofol on LPS-induced microglial activation at different time points.**

Primary mouse microglia were stimulated by LPS, followed by propofol treatment for 12, 24, 48, 96 hours. (**A**) The transcript levels of pro-inflammatory genes *Tnf* and *Nos2* in microglia were determined by RT-qPCR. (**B**) The transcript levels of NF-κB signaling component genes *Ticam1*, *Myd88*, *Irf3*, and *Nfkb1* in microglia were determined by RT-qPCR. Data were represented as mean±s.d. from three independent experiments. **, ***, and **** denote *p* < 0.01, *p* < 0.001, and *p* < 0.0001, respectively, in comparison to control group. #, ##, ###, and #### denote *p* < 0.05, *p* < 0.01, *p* < 0.001, and *p* < 0.0001, respectively, in comparison to LPS group. PPF: propofol.


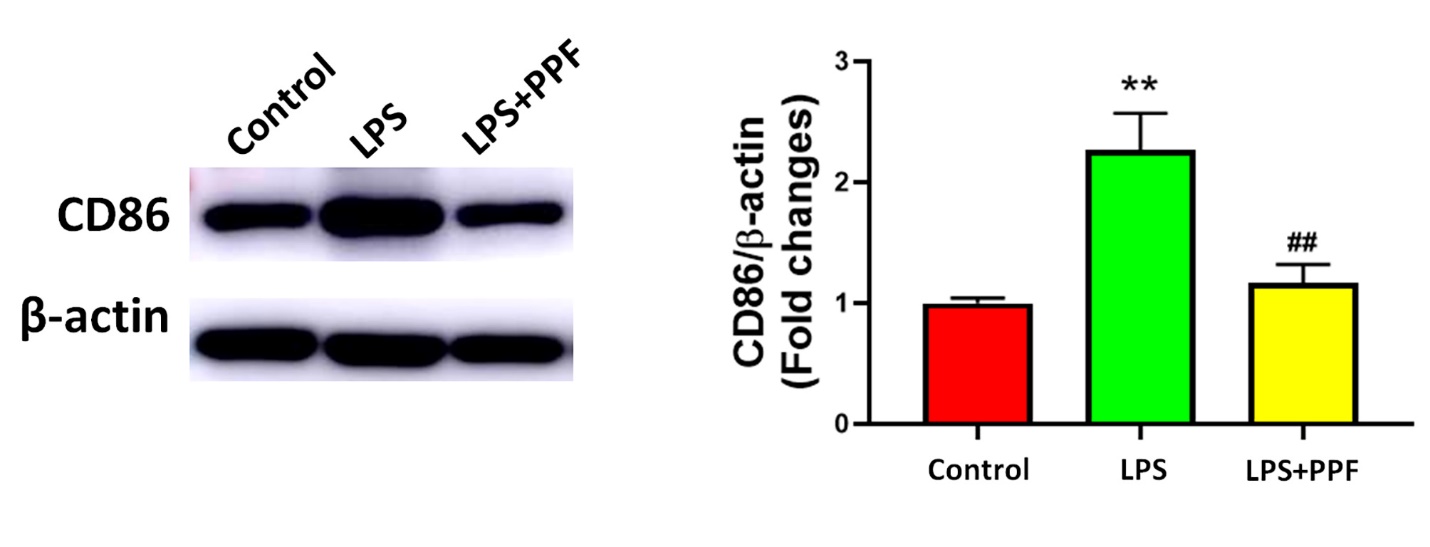


**Supplementary Figure 7. Propofol inhibits LPS-induced CD86 expression**.

The representative western blots showing the expression of CD86 in control, LPS, and LPS+PPF groups. Densitometric quantifications were presented on the right. Data were represented as mean ± s.d. from three independent experiments. ** denotes *p* < 0.01 in comparison to control. ## denotes *p* < 0.01 in comparison to LPS group. PPF: propofol.


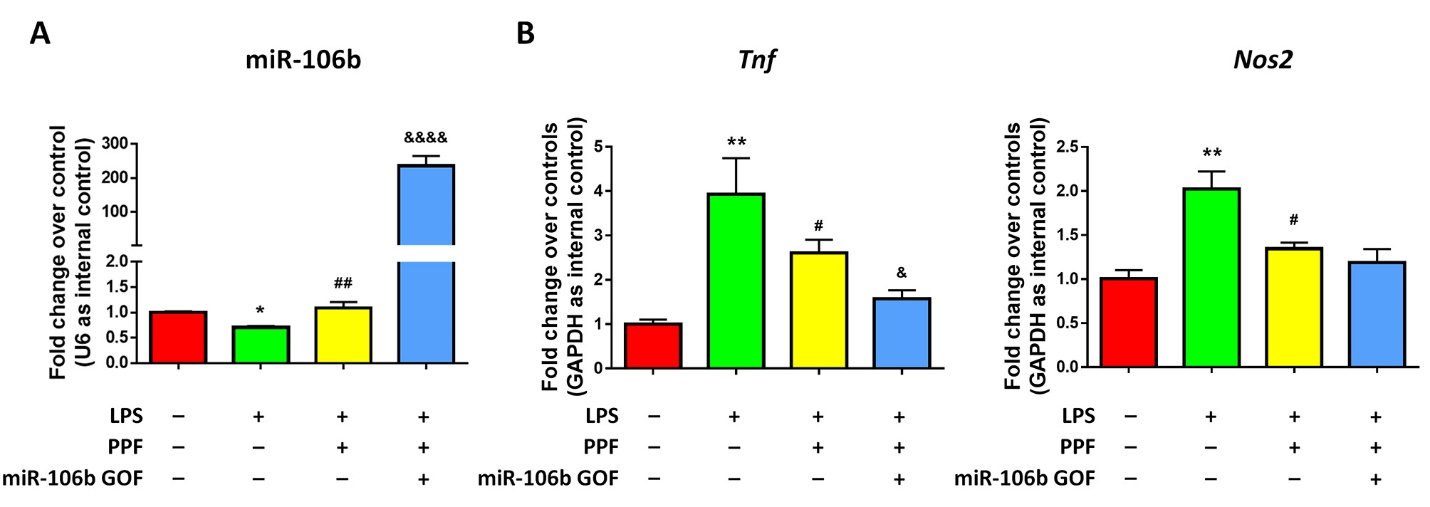


**Supplementary Figure 8. miR-106b participates propofol-mediated regulation of microglial activation**.

Primary mouse microglia were stimulated by LPS, followed by propofol treatment and the transfection of either miR-106b mimics or mimics control for 2 days. (**A**) The expression of miR-106b in microglia were determined by RT-qPCR. (**B**) The transcript levels of pro-inflammatory genes Tnf and Nos2 in microglia were determined by RT-qPCR. Data were represented as mean±s.d. from three independent experiments. *, ** and **** denote *p* < 0.05, *p* < 0.01 and *p* < 0.0001, respectively, in comparison to control group. #, ##, and #### denote *p* < 0.05, *p* < 0.01, and *p* < 0.0001, respectively, in comparison to LPS group. & and &&&& denote *p* < 0.05 and *p* < 0.0001, respectively, in comparison to LPS+PPF group. PPF: propofol. GOF: gain-of-function.


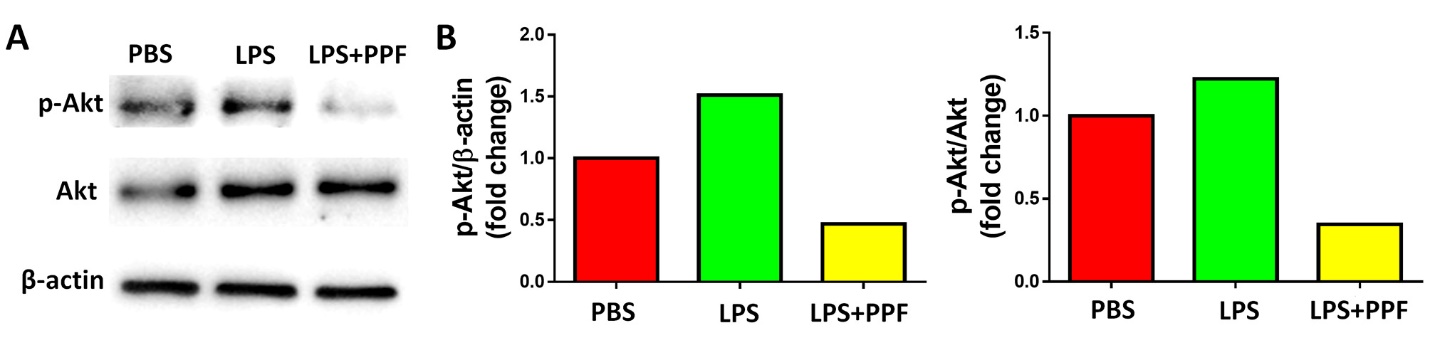


**Supplementary Figure 9. Propofol inhibits LPS-induced Pi3k/Akt pathway activation**.

(**A**) The representative western blots showing the expression of p-Akt and Akt in. (**B**) Densitometric quantifications of the signal intensity. PPF: propofol.


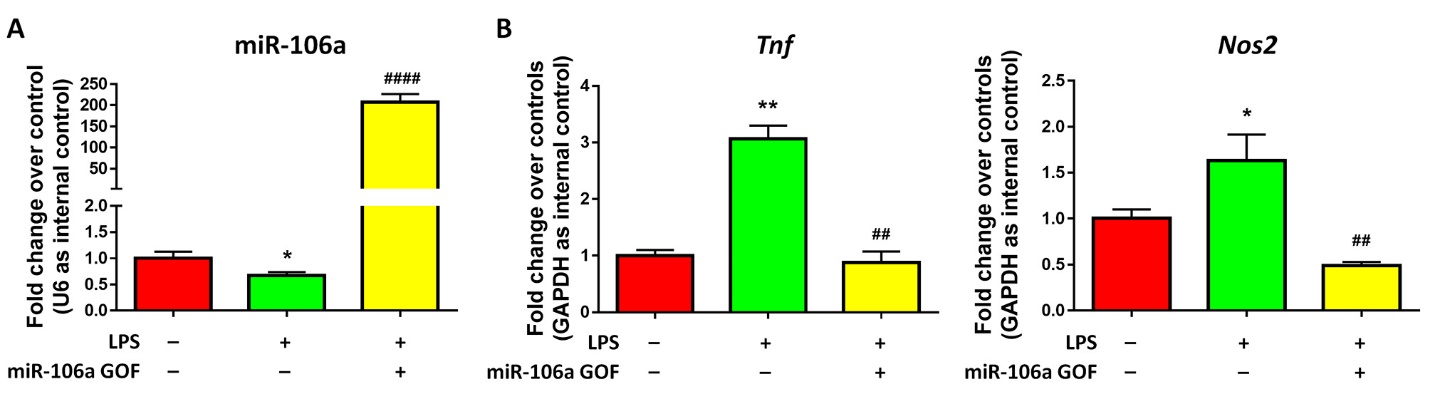
 **Supplementary Figure 10. miR-106a inhibits LPS-induced microglial activation**.

Microglia were treated with LPS, followed by the transfection of either miR-106a mimics or mimics control for 2 days. (**A**) The overexpression of miR-106a were determined by RT-qPCR, validating the transfection. (**B**) The expression levels of *Tnf* and *Nos2* transcripts were determined by RT-qPCR. Data were represented as mean±s.d. from three independent experiments. *, **, and **** denotes *p* < 0.05, *p* < 0.01, and *p* < 0.0001 in comparison to control. ## and #### denote *p* < 0.01 and *p* < 0.0001 in comparison to LPS group. GOF: gain-of-function.


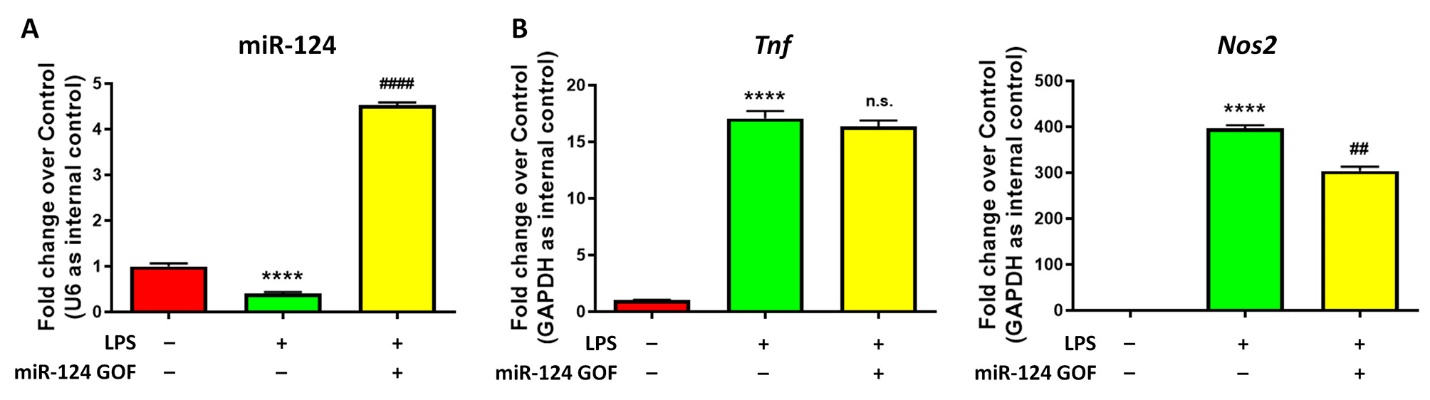


**Supplementary Figure 11. miR-124 has mild effects on microglial activation**.

Microglia were treated with LPS, followed by miR-124 mimics transfection for 2 days. (**A**) The expression levels of miR-124 were determined by RT-qPCR, validating the transfection. (**B**) The expression levels of *Tnf* and *Nos2* transcripts were determined by RT-qPCR. Data were represented as mean±s.d. from three independent experiments. **** denotes *p* < 0.0001 in comparison to control. ## and #### denote *p* < 0.01 and *p* < 0.0001 in comparison to LPS group. GOF: gain-of-function.

| Gene | Sequence | Size (bp) | T^o^ | Accession N. |
| --- | --- | --- | --- | --- |
| *Irf3* | 5’-AAGGAGTACGTGGGGCAGGT-3’  5’-TGTAGCGTGGGGAGTGTCCA-3’ | 256 | 57 | NM_016849.4 |
| *Gapdh* | 5’-CATGTTCCAGTATGACTCCACTC-3’  5’-GGCCTCACCCCATTTGATGT-3’ | 136 | 60 | NM_001289726.1 |
| *MyD88* | 5’-CGGGTCCCTGGACTCCTTCA-3’  5’-CGCGTTTCCAGCTCTCGGAT-3’ | 171 | 57 | NM_010894.2 |
| *Nfbk1* | 5’-CCTCTCTCGTCTTCCTCCAC-3’  5’-GTTTGCGGAAGGATGTCTCC-3’ | 94 | 57 | NM_008689.2 |
| *Nos2* | 5’-CCCTTCAATGGTTGGTACATGG-3’  5’-ACATTGATCTCCGTGACAGCC-3’ | 158 | 57 | NM_001313922.1 |
| *Tnf* | 5’-ACGTGGAACTGGCAGAAGAG-3’  5’-GGTCTGGGCCATAGAACTGA-3’ | 206 | 57 | NM_013693.3 |
| *Ticam1* | 5’-TGGGCTCCCACAGTGGCTAT-3’  5’-GTGCCTCTGGCCCCATCATC-3’ | 168 | 56 | NM_174989.4 |
| miRNA |  |  |  |  |
| Universal primer | 5’-GAATCGAGCACCAGTTACGC-3’ |  |  |  |
| U6 | 5’-TGGCCCCTGCGCAAGGATG-3’ |  | 55 |  |
| let-7b | 5’-TGAGGTAGTAGGTTGTGTGGTT-3’ |  | 55 | MIMAT0000522 |
| miR-10b | 5’-TACCCTGTAGAACCGAATTTGTG-3’ |  | 55 | MIMAT0000208 |
| miR-101a | 5’-TACAGTACTGTGATAACTGAA-3’ |  | 55 | MIMAT0000133 |
| miR-106a | 5’-CAAAGTGCTAACAGTGCAGGTAG-3’ |  | 55 | MIMAT0000385 |
| miR-106b | 5’-TAAAGTGCTGACAGTGCAGAT-3’ |  | 55 | MIMAT0000386 |
| miR-124a | 5’-TAAGGCACGCGGTGAATGCC-3’ |  | 55 | MIMAT0000716 |
| miR-125a | 5’- TCCCTGAGACCCTTTAACCTGTGA-3’ |  | 55 | MIMAT0000135 |
| miR-126-3p | 5’- CGCGTACCAAAAGTAATAATGTG-3’ |  | 55 | MIMAT0029895 |
| miR-127 | 5’- TCGGATCCGTCTGAGCTTGGCT-3’ |  | 55 | MIMAT0000139 |
| miR-130a-3p | 5’-CAGTGCAATGTTAAAAGGGCAT-3’ |  | 55 | MIMAT0000141 |
| miR-133a-3p | 5’-TTTGGTCCCCTTCAACCAGCTG-3’ |  | 55 | MIMAT0000145 |
| miR-141 | 5’-TAACACTGTCTGGTAAAGATGG-3’ |  | 55 | MIMAT0000153 |
| miR-142a-3p | 5’-TGTAGTGTTTCCTACTTTATGGA-3’ |  | 55 | MIMAT0000155 |
| miR-143 | 5’-TGAGATGAAGCACTGTAGCTC-3’ |  | 55 | MIMAT0000247 |
| miR-144-3p | 5’-TACAGTATAGATGATGTACT-3’ |  | 55 | MIMAT0000156 |
| miR-145a-5p | 5’-GTCCAGTTTTCCCAGGAATCCCT-3’ |  | 55 | MIMAT0000157 |
| miR-146a-5p | 5’-TGAGAACTGAATTCCATGGGTT-3’ |  | 55 | MIMAT0000158 |
| miR-15a-5p | 5’-TAGCAGCACATAATGGTTTGTG-3’ |  | 55 | MIMAT0000526 |
| miR-153-3p | 5’-TTGCATAGTCACAAAAGTGATC-3’ |  | 55 | MIMAT0000163 |
| miR-155 | 5’-TTAATGCTAATTGTGATAGGGGT-3’ |  | 55 | MIMAT0000165 |
| miR-16-5p | 5’-TAGCAGCACGTAAATATTGGCG-3’ |  | 55 | MIMAT0000527 |
| miR-17 | 5’-CAAAGTGCTTACAGTGCAGGTAG-3’ |  | 55 | MIMAT0000386 |
| miR-18a-5p | 5’-TAAGGTGCATCTAGTGCAGATAG-3’ |  | 55 | MIMAT0000528 |
| miR-181c | 5’-AACATTCAACCTGTCGGTGAGT-3’ |  | 55 | MIMAT0000674 |
| miR-182-5p | 5’-TTTGGCAATGGTAGAACTCACACCG-3’ |  | 55 | MIMAT0000211 |
| miR-183 | 5’-TATGGCACTGGTAGAATTCACT-3’ |  | 55 | MIMAT0000212 |
| miR-185-5p | 5’-TGGAGAGAAAGGCAGTTCCTGA-3’ |  | 55 | MIMAT0000214 |
| miR-19a-3p | 5’-TGTGCAAATCTATGCAAAACTGA-3’ |  | 55 | MIMAT0000651 |
| miR-192 | 5’-CTGACCTATGAATTGACAGCC-3’ |  | 55 | MIMAT0000517 |
| miR-193a-3p | 5’-AACTGGCCTACAAAGTCCCAGT-3’ |  | 55 | MIMAT0000223 |
| miR-195a-5p | 5’-TAGCAGCACAGAAATATTGGC-3’ |  | 55 | MIMAT0000225 |
| miR-200b-3p | 5’-TAATACTGCCTGGTAATGATGA-3’ |  | 55 | MIMAT0000233 |
| miR-203b-3p | 5’-GTGAAATGTTTAGGACCACTAG-3’ |  | 55 | MIMAT0000236 |
| miR-204-5p | 5’-TTCCCTTTGTCATCCTATGCCT-3’ |  | 55 | MIMAT0000237 |
| miR-21a-5p | 5’-TAGCTTATCAGACTGATGTTGA-3’ |  | 55 | MIMAT0000530 |
| miR-216a-5p | 5’-TAATCTCAGCTGGCAACTGTGA-3’ |  | 55 | MIMAT0000662 |
| miR-22 | 5’-AAGCTGCCAGTTGAAGAACTGT-3’ |  | 55 | MIMAT0000531 |
| miR-23b | 5’-ATCACATTGCCAGGGATTACC-3’ |  | 55 | MIMAT0000125 |
| miR-25-3p | 5’-CATTGCACTTGTCTCGGTCTGA-3’ |  | 55 | MIMAT0000652 |
| miR-26a-5p | 5’-TTCAAGTAATCCAGGATAGGCT-3’ |  | 55 | MIMAT0000533 |
| miR-27a-3p | 5’-TTCACAGTGGCTAAGTTCCGC-3’ |  | 55 | MIMAT0000537 |
| miR-29b-3p | 5’-TAGCACCATTTGAAATCAGTGTT-3’ |  | 55 | MIMAT0000127 |
| miR-30a | 5’-TGTAAACATCCTCGACTGGAAG-3’ |  | 55 | MIMAT0000128 |
| miR-30c-1-3p | 5’-CTGGGAGAGGGTTGTTTACTCC-3’ |  | 55 | MIMAT0000416 |
| miR-302c-3p | 5’-AAGTGCTTCCATGTTTCAGTGG-3’ |  | 55 | MIMAT0003376 |
| miR-31-5p | 5’-AGGCAAGATGCTGGCATAGCTG-3’ |  | 55 | MIMAT0000538 |
| miR-323-3p | 5’-CACATTACACGGTCGACCTCT-3’ |  | 55 | MIMAT0000551 |
| miR-34a-5p | 5’-TGGCAGTGTCTTAGCTGGTTGT-3’ |  | 55 | MIMAT0000542 |
| miR-340-5p | 5’-TTATAAAGCAATGAGACTGATT-3’ |  | 55 | MIMAT0004651 |
| miR-409 | 5’-GAATGTTGCTCGGTGAACCCCT-3’ |  | 55 | MIMAT0001090 |
| miR-7a | 5’-TGGAAGACTAGTGATTTTGTTGT-3’ |  | 55 | MIMAT0000677 |
| miR-9 | 5’-TCTTTGGTTATCTAGCTGTATGA-3’ |  | 55 | MIMAT0000142 |
| miR-93-5p | 5’-CAAAGTGCTGTTCGTGCAGGTAG-3’ |  | 55 | MIMAT0000540 |
| miR-96 | 5’-TTTGGCACTAGCACATTTTTGCT-3’ |  | 55 | MIMAT0000541 |

**Supplementary Table 1. List of specific primers.**

**Materials and Methods**

**Immunocytochemistry**

Enriched primary microglia were fixed in 4% formaldehyde for 20 minutes at room temperature (RT) and then washed with PBS for three times. The fixed microglia were permeabilized with 0.2% Triton X-100 in PBS for 10 minutes, blocked with 2% BSA in PBS for 1 hour at RT, and incubated overnight at 4°C with primary antibodies including anti-Iba-1 (Cat # 019-19741, WAKO; Cat # AB5607, Abcam), anti-Nestin (Cat # NB100-1604, NOVUS), anti-Tuj1 (Cat # T8860, Sigma), anti-Map2 (Cat # MB0078, Bioworld), anti-Gfap (Cat # AB5541, Millipore), anti-Eaat1 (Cat # AB416, Abcam), and anti-O4 (Cat # O7139, Sigma). Primary antibodies were washed away by PBS for three times and cells were incubated for 1 hour at RT with secondary antibodies including anti-rabbit IgG (coupled with Alexa Fluor 568, Life Technologies), anti-goat IgG (coupled with Alexa Fluor 568, Life Technologies), anti-rabbit IgG (coupled with Alexa Fluor 488, Life Technologies), anti-chicken IgG (coupled with Alexa Fluor 488, Life Technologies), and anti-mouse IgG (coupled with Alexa Fluor 488, Life Technologies). Cells were mounted using VectaShield (Vector Laboratories, Burlingame, CA) and images were taken by a Zeiss AX10 fluorescence microscope accompanied with ZEN 2.3 (blue edition) software.

**TUNEL staining**

The cultured cells were planted on coverslips and fixed in 4% formaldehyde for 20 minutes at RT. Terminal deoxynucleotidyl transferase-mediated dUTP nick end labeling (TUNEL) assay was then performed using TUNEL assay kit (Promega, G3250) according to the manufacturer’s instruction. Images were taken by a Zeiss AX10 fluorescence microscope accompanied with ZEN 2.3 (blue edition) software. For quantification of the percentage of specific cell types in each experiment, cell type-specific antigen positive cells were counted from 15 random fields per group in three coverslips (5 fields each).

**CCK8 assay**

Briefly, 5000 cells/well microglia were pre-treated with LPS (50 ng/ml) for 2 hours and then treated with different doses of propofol for 2 days on 96-well plates. Cell viability was measured by CCK-8 (yeasen, #40203ES80) assays. Experiments were handled according to the manufacturer's instructions. Absorbance was measured at 450 nm and analyzed using SpectraMax M5 microplate readers (Molecular Devices).
